# Nutrition and Female Fertility: An Interdependent Correlation

**DOI:** 10.3389/fendo.2019.00346

**Published:** 2019-06-07

**Authors:** Erica Silvestris, Domenica Lovero, Raffaele Palmirotta

**Affiliations:** Department of Biomedical Sciences and Human Oncology, University of Bari Aldo Moro, Bari, Italy

**Keywords:** obesity, infertility, anovulation, oocytes, diet, nutrition

## Abstract

Besides aging, a number of non-modifiable lifestyle-related factors, such as smoking, elevated consumption of caffeine and alcohol, stress, agonist sports, chronic exposure to environmental pollutants, and other nutritional habits exert a negative impact on a women's fertility. In particular, metabolic disorders including diabetes, obesity, and hyperlipidemia commonly associated to hypercaloric diets are suspected to affect a woman's fertility either by direct damage to oocyte health and differentiation, or by indirect interference with the pituitary-hypothalamic axis, resulting in dysfunctional oogenesis. Obese women show decreased insulin sensitivity determining persistent hyperinsulinemia, which may be involved in the pathogenesis of Polycystic Ovary Syndrome. Thus, the reduced insulin secretion induced by dietary adjustments is an attractive non-pharmacological treatment to prevent infertility, and a Mediterranean diet aimed at maintaining normal body mass may be effective in the preservation of ovarian health and physiology. Furthermore, in relation to the oxidative stress as a co-factor of defective oocyte maturation, an appropriate intake of proteins, antioxidants and methyl-donor supplements (1-Carbon Cycle) may decrease the bioavailability of toxic oxidants resulting in the protection of oocyte maturation.

## Introduction

Infertility is a major problem in modern society and recurs in as much as 20–30% of the fertile female population. The American Society of Reproductive Medicine (ASRM) delineates infertility as the failure to conceive after one or more years of attempts of natural fertilization (1), with the World Health Organization (WHO) reporting up to 80 million women world-wide having been affected by this disease to date, with a prevalence of ~50% of all women in developing countries (2).

Besides a number of gynecological and systemic diseases affecting a woman's fertility, lifestyle factors and environmental conditions such as stressful jobs, unbalanced nutrition and unhealthy diet concur to interfere with reproduction safety in both women and men. Therefore, abnormal body weight and energy supply in terms of restrictions or excesses, as well as dietary enrichment in carbohydrates, fatty acids, proteins, vitamins and minerals all exert a detrimental impact on both ovulatory function and normal spermatogenesis. In addition to the negative interference with the safety of gametes, several nutrients of major diets also affect the implantation of a normal embryo.

In this paper, we revise how several lifestyles and rough nutritional regimens may interact with the reproductive health in women and how adequate nutritional support may improve fertility, according to studies from different clinical investigators and from our own observation.

## Major Lifestyle Factors Affecting Women's Fertility

Beyond age, a number of lifestyle-related factors, such as excess body weight, obesity, smoking, intense sporting activity, alcohol consumption, drug addiction or abuse of other substances, have an adverse influence on female fertility. To date, most infertilities are usually treatable with major procedures belonging to the Assisted Reproductive Technology (ART) methods. However, the normalization of those factors could probably restore normal oocyte maturation and prevent the adoption of these procedures (3). Therefore, the major factors influencing infertility are listed and commented on in detail below.

### Age

Besides lifestyle factors, woman's age is a major factor influencing the spontaneous probability of conception that already starts to decrease by 25–30 years of age. Given the age-correlated deterioration of the ovarian reserve and oocyte quality, it is expected that the global trend of postponing maternity will result in increasing involuntary childlessness.

The improvement of oocyte vitrification resulting in pregnancy and live birth rate increases comparable to using fresh oocytes, which has offered a chance to cryopreserve oocytes for upcoming practice, presenting women with the possibility to delay their motherhood (4).

### Tobacco Smoking, Coffee and Alcohol Abuse

Tobacco smoking markedly affects the reproductive health of both men and women, albeit acting in different ways. Heavy alcohol consumption indirectly affects the fertility when associated with nutritional or secondary health disorders (5). In females, smoking is associated with a rapid decline of ovarian reserves, delayed conception and heightened risk of spontaneous miscarriage, as well as a lower success rate from ART, while in males the percentage of normal semen morphology and motility is significantly reduced (6, 7). The influence of alcohol intake on reproductive outcomes has already been investigated in several studies, yet it is still impossible to demonstrate a significant correlation between alcohol intake and oocyte maturation and fertilization in females, and between male alcohol consumption and the rate of lost pregnancies (8, 9).

Firns and co-workers evaluated the impact of these lifestyle factors on *in vitro* fertilization (IVF) outcomes in 351 couples attending the PIVET Medical Center in Western Australia (5). Considering the quantity of collected oocytes, fertilization rates, pregnancy, and pregnancy loss, the multiple regression analysis showed that smoking strongly damages the quality of gametes in both sexes resulting in a reduction of ovarian reserve in women, and in a significant decrees in density, count, mobility and morphology of sperm in men. On the contrary, female alcohol consumption did not show any correlation with fertility parameters, while in males it even showed a positive effect on fertilization rate, in the cohort with an associated consumption of fruit and vegetables (5). Therefore, based on the results of the Authors and the current literature, there is an important impact of tobacco smoking on IVF clinical outcomes, whereas a defined role of alcohol assumption needs to be still defined (10).

Finally, evidence suggests that high intake of caffeine has a potential dose-response association resulting in both a longer time for conception and increased risk of pregnancy loss (10).

### Stress

A stressful life, particularly in hard-working women, may contribute to cause infertility since symptoms related to anxiety and depression are described as more frequent in infertile than in fertile females. These features concur to produce a condition of psychological stress that may alter the physiological oocyte maturation (11). In a meta-analysis performed on 2,202 patients Purewal and contributors demonstrated that a favorable success of ART treatments leading to a higher rate of conception is obtainable in the absence of depression and anxious mental states (12).

Furthermore, it has been described that stress management by periodic relaxing training is helpful in decreasing the psychological distress in infertile women and may result in increased conception rates (13). In a very recent study performed on 72 women subjected to ART, Miller and coworkers demonstrated an increase in salivary cortisol levels during the pretreatment phase, followed by a reduction at the time of the embryo transfer. They, thus, concluded that the effects of both physiological and psychological stress on IVF outcome could not be always negative (14).

### Nutrition Styles and Body Weight

Reproductive performance is definitely influenced by foods and type of nutrition. An unbalanced caloric and protein intake due to incorrect food consumption, responsible for severe under- or over-weight, leads to alterations of the ovarian function with subsequent increase in the infertility. Several studies exploring the effect on fertility of various dietary habits are based on data from extended studies including 116,678 females in the Nurses' Health Study II, that defined the reduced risk of fertility due to ovulatory disorder in women whose food regimen included low glycemic content and limited intake of nutrients (15). Variations of the body weight in terms of overweight, obesity or severe underweight associated to alterations of the energy balance are also suspected to produce ovulatory disorders. To this regard, it has been reported that the time to conceive is longer in women with body mass index (BMI) superior to 25 kg/m^2^ or inferior to 19 kg/m^2^, and that both overweight and obesity are significantly related with reduced pregnancy rate, increased supplies of gonadotrophins and higher miscarriage rate. High BMI is also associated with adverse pregnancy outcomes such as gestational diabetes, hypertension and premature births and unbalanced diets with a prevalence of carbohydrates, fatty-acids, proteins or vitamins and micronutrients definitely exert a negative impact on ovulation. Moreover, nutritional factors may influence not only oocyte maturation, but also quality of embryos and efficiency of implantation. However, more information regarding the role of nutrition in procreation is needed to provide guidelines devoted to nutritional management of infertile women (15).

### Environmental Pollutants

The Occupational Safety and Health Administration (OSHA) postulated that long-lasting exposure to chemical agents as organic solvents, heavy metals, aromatic amines, pesticides and vegetal toxins is related to reduced fertility and improved predisposition to occasional or recurrent miscarriages. Furthermore, environmental pollutants, determining the formation of DNA adducts and abasic sites construction, can induce DNA modifications in gametes and embryos by introducing genetic mutations once unrepaired (16).

## Nutritional Disorders

A normal reproductive performance definitely requires a healthy nutrition since malnourished males and females are reported as major infertile populations in developing countries while, eating in excess, fast food consumption, hypercaloric dietary regimens and obesity, concur to infertility in well-developed and western societies.

Particularly in women, abnormal nutrition may permanently affect oocyte maturation, and a better understanding of the molecular events deranged in malnourished people would provide solutions for restoring normal reproductive functions.

### Malnutrition

Deficient food intake, inadequate alimentary regimes, strong dietary restrictions and a general lack of nutrients result in loss of both body weight and physical performance, delayed puberty, lengthening of the post-partum interval to conception, lower gonadotropin secretion levels with alterations of the physiological ovarian cyclicity and increased infertility. Poor intake of proteins, micro and macro-minerals and vitamins is associated with reduction in reproductive performance since the altered energy balance is directly correlated to the reduced ovulatory maturation in women (17). Thus, inadequate nutrition is closely linked to female reproductive pathophysiology. This is confirmed by the fact that both bulimia nervosa and anorexia, namely two pathologic conditions affecting 5% of women in childbearing age, are indisputable causes of amenorrhea, infertility and miscarriages (18).

### Overweight and Obesity

Overweight and obesity are diffused pathological conditions during the woman's reproductive age, with an incidence up to 20–25% among patients consulting for infertility. WHO estimates that 9% to 25% of females in industrialized countries are obese and at greater risk of generating obese children, particularly when affected by gestational diabetes. Through insulin resistance (IR) and high levels of insulin and androgens, the adipose tissue is responsible for ovulatory disorders in disposed patients and the anovulation associated to obesity is responsible for higher risk of miscarriages and infertility (19). Management of anovulation in obese women includes diet and exercise in parallel with standard methodologies of ovulation induction.

In patients without ovulatory disorders, overweight and obesity extend the time to conceive, decrease the outcome of infertility treatment and increase the rates of gestational diabetes, hypertension, cesarean section, overweight newborns, perinatal mortality and morbidity. A number of nutritional and clinical studies have confirmed that the Mediterranean Dietary (MedDiet) patterns and regular physical activity in overweight women significantly reduce unsuccessful attempts to conceive and improve the efficacy of ARTpregnancy programs. Therefore, the evaluation of lifestyle habits and the modification of unhealthy behaviors by appropriate assistance or with specific management, such as acid folic supplementation, must be systematic in females attempting to conceive (20).

## Nutritional Related Diseases

### Obesity and Anovulation

IR and hyperinsulinemia are the primary underlying metabolic abnormalities reported in obesity, which are key features of the Metabolic Syndrome (MS) and PCOS, and have a significant impact on female fertility. Elevated circulating insulin levels and IR provide an unfavorable biochemical environment in the ovaries, increasing both androgen over-synthesis and lipid metabolism from theca cells, that in turn induce a central distribution of fat and dyslipidemia. The android fat distribution pattern may be justified by hyperandrogenism, resulting in a vicious circle of central adiposity, hyperinsulinism, and metabolic aberrations (21). Also, cytokines are highly active in ovarian physiology, where they are involved in the formation of a favorable environment for the selection and growth of the follicle since these molecules are involved in key processes of cell proliferation and differentiation, survival and atresia of the follicle and oocyte development (22). Several cytokines, such as TGF-β-superfamily members, are involved in all steps of folliculogenesis while the production of others is stage-dependent: FGF-2 and VEGF, together with SDF-1a and leptin, are also capable of regulating corpus luteum development and function. In particular, leptin stimulates estrogen synthesis in luteinized granulosa cells and reduces progesterone synthesis in insulin stimulated theca cells. This mechanism also explains infertility related to obesity, an altered metabolic state in which the concentrations of leptin are particularly high (23).

### Polycystic Ovary Syndrome (PCOS)

PCOS is a recurrent reproductive endocrinological disorder that affects up to 20% of women of reproductive age globally (24). Basic diagnostic criteria for PCOS include at least two of the following criteria: oligo-anovulation, hyperandrogenism and ultrasound-ascertained polycystic morphology of one ovary (minimum 12 follicles of 2–9 mm in diameter or ≥10 cm^3^ ovarian volume) (24). The IR and hyperinsulinemia play a significant role in the development of PCOS and contribute to the development of MS (25). However, the mechanism by which hyperinsulinemia and hyperandrogenism are responsible for the deregulation of ovarian function is unclear. Experimental data show that through its receptor, insulin has specific activities on steroidogenesis by stimulating the cells of the theca to oversecrete androgens and improving the responsiveness of ovaries to the luteinizing hormone (LH) for the androgen production (26).

**Figure 1:**
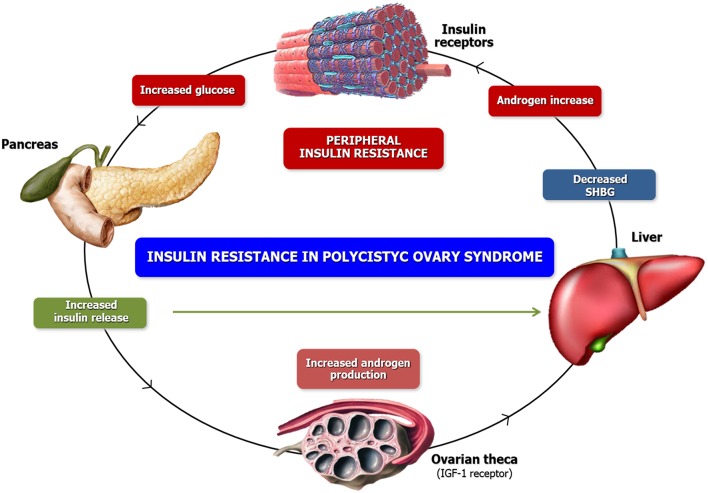
Phatogenesis of Hyperandrogenism. Similar to the high bio-availability of androgens, high insulin concentrations inhibit SHBG production. However, the combined activity of insulin and androgens reduces the SHGB concentrations yielding increased free androgen levels which aggravate the underlying insulin resistance. These conditions ultimately foster a self-propagating positive feed-back loop that increases in severity over time. On the other side, insulin stimulates ovarian androgen production acting via insulin receptors on theca/interstitial cells in ovarian stroma. At high levels, insulin also binds to IGF-1 receptors or possible hybrid receptors, which are structurally similar and use a similar signaling mechanism.

The insulin-like growth factor-I receptor (IGF-IR) could also be involved in the mechanism of hyperandrogenism induced by hyperinsulinemia observed in PCOS patients. In fact, when bound to IGF-IR, the insulin activates β-subunit tyrosine kinase activity by amplifying the normal IGF-I signal (27). Moreover, the insulin-like growth factor binding proteins (IGFBPs) are involved in the systemic and local regulation of IGF activity by both IGFI and IGFII with high affinity. These complexes thus lead to a reduction in the hepatic secretion of insulin growth-factor-binding protein-I (IGFBP-I) and a consequent bioavailability of IGF-I, with the final effect of enhancing the androgen production by both interstitial and stromal cells of the theca (23) (Figure 1).

Previous studies have established that patients affected by PCOS with BMI in the highest quartile (≥30 Kg/m^2^) have a very high risk of developing MS (28). PCOS is the major cause of infertility due to anovulatory disorders although the anovulation mechanisms are unclear. The anovulation is characterized by an apparent arrest of antral follicle development at the 5–10 mm developmental stage thus implying a failure in entering the cycle preovulatory phase. Under specific conditions, however, the spontaneous ovulation may also occasionally occur, and the disorder can be reverted in most cases by increasing the FSH serum levels.

PCOS is often accompanied with altered gonadotropin levels, decreased levels of IGF-BP1, increased ovarian 17-hydroxiprogesterone (17-OHP) and androgen response to Gonadotropin-Releasing-Hormone agonists (GnRH-agonists). PCOS affects about 75% of women suffering from anovulation-related infertility and its management depends on symptoms. Thus, appropriate therapy for PCOS becomes necessary in order to induce ovulatory cycles and fertility (28).

Metformin is the most commonly used insulin-sensitizing drug to treat PCOS thanks to its ability to increase insulin sensitivity, reduce hepatic gluconeogenesis, as well as to inhibit hepatic uptake of lactate and alanine, and to increase the conversion of pyruvate to alanine. The molecular pathway triggered by Metformin is today partially unclear. Several studies have identified multiple potential mechanisms of action such as the inhibition of the mitochondrial respiratory chain and glycerophosphate dehydrogenase, hyperactivation of AMP-activated protein kinase (AMPK) or cyclic adenosine monophosphate (cAMP), inhibition of glucagon and deregulation of gut microbiota (29). These mechanisms, together or individually, result in an increase of oocyte maturation and a favorable effect on infertility in PCOS. A lower-carbohydrate diet capable of inducing a reduction in insulin production may be a valid-alternative to the drug regimen. In fact, the decrease of carbohydrate uptake downregulates the glucose stimulus to pancreatic β-cells reducing the amount of secreted insulin. While several studies have explored the correlations between diet quality and weight loss (30), other observations have investigated in weight-stable conditions the possible consequence of diet composition on β-cell responsiveness and insulin sensitivity (31, 32).

### Metabolic Syndrome (MS)

MS is a very common disease in Western countries and includes multiple endocrine disturbances, such as overweight, altered levels of hepatocytolysis, arterial hypertension, obesity, dyslipidemia, and IR. MS is a major social health problem, particularly in developed nations such as the United States but also in Europe, with a prevalence of 20 and 30%, respectively (33). Although not completely assessed, several factors have been implicated and primarily include the hypercaloric diet in association with deregulated dietary habits, sedentary lifestyles, increased age and augmented BMI. MS is also suspected to play a definite role in carcinogenesis, particularly in the gastrointestinal tract.

Several studies have demonstrated that females with MS, inadequate metabolic control and primary or secondary amenorrhea show low levels of LH and FSH, associated with a lack of residual insulin secretion (34, 35). Such studies have demonstrated abnormalities of GnRH pulse generator, as well as a decrease in numbers and amplitude of LH pulses in patients with diabetes and amenorrhea compared to patients with normal menstrual cycles. On the other hand, IR, hyperinsulinemia and related metabolic abnormalities in MS may exert a role in the progress of the PCOS (36).

Interestingly, all therapeutic approaches used for the correction of insulin homeostasis in obese and MS patients, such as Thiazolidinediones, Metformin, lifestyle modification for weight reduction or bariatric surgery have been proven to produce restoring effects on ovulation and hyperandrogenemia (37).

## Healthy Nutrition for a Healthy Ovulation

Nutrition plays a major role in enhancing the reproductive efficiency both in women and men, and contrarily to the detrimental role of body weight, the effect of the diet in female fertility is not well-defined. However, the interaction between nutrition and fertility appears critical for the reproductive performance and the relationship between ovulatory disorders and metabolic diseases such as diabetes and/or galactosemia suggests that dietary factors exert etiological role in some variants of infertility (34).

A study performed in 2006 on 12,579 subjects from the Southampton Women's Survey has demonstrated that female and male preconceptional nutritional status influences fertility and perinatal conditions (35, 38).

This observational prospective study performed on 161 women from the Food, Lifestyle and Fertility Outcome Project has revealed that a diet rich in fish, legumes, vegetables and low in carbohydrates was linearly related with red blood cell folate and vitamin B6 in blood and follicular fluid, with a 40% increase in the chance of pregnancy by IVF intracytoplasmic sperm injection (ICSI) (36). Similarly, a dietary intake of Polyunsaturated omega-3 fatty acids (FAs), Alpha-linolenic acid and Docosahexaenoic acid (DHA) is related to a positive outcome in women undergoing IVF/ICSI (37).

A case-control study on 485 women from the Seguimiento Universidad de Navarra (SUN) Project suggested that reproductive outcomes were improved in couples with a correct balanced diet (39). Although some associations have been identified between periconceptional diet and fertility, the mechanism by which nutritional status can influence implantation, embryo quality and perinatal or child health still remains undefined (40).

The role of different nutrients that could have an impact on female fertility are summarized in the following sections.

### Proteins

The role of protein intake on reproduction is complex and it is still unclear how the source or the amount of protein consumption may affect the ovulatory function or women's fertility. However, it is well-known that protein intake has been associated with a deregulation of the steroidogenesis in women affected by PCOS, likely by reducing hyperinsulinemia. To this regard, Mumford et al. demonstrated that in healthy women a protein-rich diet, particularly of animal proteins, is significantly associated with lower testosterone levels, thus highlighting the potential correlation between protein intake and androgen synthesis (41).

Furthermore, in a cohort of healthy women Chavarro and coworkers, showed that the consumption of animal or vegetable proteins was associated with increased or lower risk of ovulatory infertility, respectively. This correlation is statistically significant in women older than 32 years but the underlying mechanisms remain unclear (42).

### Carbohydrates

To date it is still not well-established whether carbohydrate consumption could have an effect on ovulatory function and in general on fertility in healthy women. In a population of 17,544 women, Chavarro and coworkers showed that the chronic intake of carbohydrates was positively associated with ovulatory disorders (43). In this context, restored ovulatory function and fertility were observed in healthy women with PCOS by improving glucose homeostasis. Thus, it is possible that several ovulatory disorders are caused by the effects of carbohydrate intake on glucose metabolism (44). Therefore, a higher dietary glycemic load appears related with elevated fasting glucose levels, hyperinsulinemia and IR, that are responsible for a higher release of free IGF-I and androgens ultimately resulting in endocrine disturbance and oocyte maturative defects (45).

### Lipids

The impact of fats on reproduction in women is an actual focus of investigative research. Dietary fatty acids and cholesterol intakes are theorized to affect fertility and pregnancy outcomes, likely through the increased production of prostaglandins and steroids (46). However, few data are available about the relationship between fat intake, androgen levels and ovulation. Mumford and collaborators observed in a group of 259 regularly menstruating women that the total fat intakes of polyunsaturated fatty acids (PUFA) were not associated with higher testosterone levels, but rather with progesterone elevations promoting a decreased risk of anovulation (47).

Therefore, their results suggest a weak role for fatty acids, specifically PUFAs, in androgen synthesis, although future studies are needed to answer the question of whether or not alterations in androgen synthesis may consistently affect the female fertility (47).

### Antioxidants

Oxidative Stress (OS) and the resulting variation of the DNA methylation are capable of impacting reproductive capacity. OS develops from a bodily imbalance between the anti-oxidant protection and free radical (ROS) release (48). Therefore, since diet is a source of exogenous oligo-elements and vitamins, current clinical practice suggests to integrate the diet with some nutritional supplements that are capable to reverse this imbalance, inducing the control of the OS and improving fertility (49). Among several existing antioxidants, Glutathione is a natural compound with a strong detoxifying activity, that maintains the redox state of the cell by limiting the production of free radicals. Other antioxidants include the Lipoic acid, Vitamin E, Vitamin C and CoenzymeQ10 (CoQ10), whose deficiency or altered concentrations, alone or in combination, may definitely impair the function of the whole detoxifying system (50).

The effect of a regular intake of ascorbic acid has been widely described in literature, showing that its consumption during pregnancy could stimulate the human placental/trophoblastic steroidogenesis that physiologically supports gestation (51). In fact, it has also been reported that among women with frequent spontaneous abortions dependent on a luteal phase defect, the blood levels of this antioxidant were lower than in females with better reproductive outcomes (52).

However, although there are many studies supporting the influence of the antioxidants intake on the reproductive capacity, less is known about their action on menstrual function (53). A report from the BioCycle Study described a positive correlation between OS and endogenous estradiol (E2) whereas no association with FSH and sex hormone-binding globulin (SHBG) is described (54). Showell and coworkers reported in their study that despite the beneficial role of antioxidants in reducing the OS, their incorrect or excessive consumption could induce some adverse effects (55) (Table 1).

**Table 1:** Natural antioxidants.

**Molecule**	**Natural source**	**Mechanism of action**	**Target organ**	**Molecular target**
Resveratrol	Red wine, peanut, grape, pine, blackberry	Anti-proliferative, anti-angiogenic, anti-inflammatory	Ovary, breast, prostate, liver, lung, stomach	P53, glutathione, AKT, NFkB, iNOS, STAT3, Survivina
Lupiol	Mango, olives, strawberry, black grapes	Anti-mutagen, anti-proliferative, anti-apoptotic	Skin, lung, pancreas, colon, prostate, liver	P21, Fas, Bcl-2, Bax, AKT, NFkB, COX-2, NOS,RAS
Betulinic acid	Widespread in the vegetable rank (birch)	Anti-inflammatory, immunomodulatory, anti-apoptotic	Skin, ovary, prostate, lung, breast, kidney, uterine cervix	PPR-g, p21, p38, NFkB, JNK, COX-2
Polynsatured Fatty acids	Corn, sunflower, olives, spinach, walnuts	Anti-apoptotic, anti-inflammatory	Colon, breast, prostate, pancreas, blood tumors	NFkB, Fas/FasL, PPr-g, Bcl-2, ERK1/2, STAT3
Glicolide B	Ginseng	Anti-angiogenic, anti-apoptotic	Ovary, brain, breast	iNOS, JNK
Luteolin	Artichoke, celery, green pepper, broccoli, mint	Anti-inflammatory, anti-allergic, anti-proliferative	Ovary, liver, colon, breast, prostate, skin, pancreas	JNK, p53, IGF-1R, EGFR, BCL-2, STAT3
Lycopene	Tomato, pink grapefruit, papaya	Anti-proliferative, anti-inflammatory, anti-angiogenic	Lung, breast, pancreas, colon	Ciclina D1, Bcl-2, AKT, NFkB

*An incomplete list with origin and molecular effects on target organs*.

Therefore, further trials are necessary to investigate the action of antioxidants on the organism in order to improve the reproductive outcome.

### Folates

It is well-known that preconceptional folic acid supplementation (400 μg per day) both improves folate and decreases homocysteine (Hcy) levels in follicular fluid. Supplementation with folic acid, or multivitamins containing folic acid, has been associated to better embryo quality, improved chances of pregnancy and reduced risk of ovulatory infertility (56). However, even if over 80% of infertile women are responsive to folic acid supplementation and the use of folic acid is more widespread among infertile women than the fertile ones, only 50% of them correctly use these products during the preconception times (57). To this regard, Murto and coworkers investigated the folate status among infertile and fertile women and showed that the first group has a significantly better folate status than the others, and that infertile patients are more prone to consume folic acid supplements than controls (58). Folates are a group of interconvertible coenzymes that play fundamental roles in DNA synthesis, methylation and protein synthesis. In fact, folate deficiency may alter these synthetic processes resulting in Hcy accumulation and consequent excessive OS and methylation reactions. The DNA methylation is an epigenetic mechanism, able to modify the expression of specific genes without changing the DNA sequence. Also, methylation alters the physical accessibility to the nucleic acids by molecular complexes responsible for gene expression and, therefore, may modify or suppress the gene function. This process is involved in numerous molecular events such as gene transcription, embryonic development, X chromosome inactivation, genomic imprinting and chromosome stability, and the methylation profile is maintained during cell division. Thus, the relative information is transmitted to the daughter cells independently of the DNA sequence (16).

**Figure 2:**
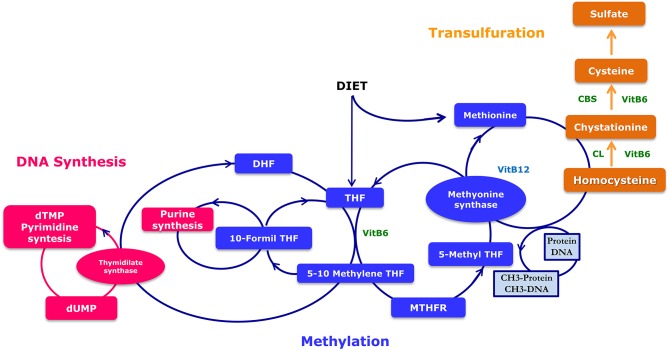
Metabolism of folates. Folic acid cycle involves the recycling of homocysteine to methionine and contains the methyltetrahydrofolate receptor (MTHFR) necessary for the formation of 5-THF. MTHFR catalyzes the reduction of methylentetrahydrofolate (5,10-methylen-THF) to methyl (5-methyl THF) by donating a methyl group. MS can catalyze the transfer of the methyl group from 5-methyl THF to homocysteine, which generates methionine and THF. The cystathionine-beta synthase pathway allows the formation of cysteine from homocysteine, that is a precursor of glutathione and hypotaurine. Impaired methylation will thus lead to a number of major genetic health problems. DHF, Dihydrofolate reductase; THF, Tetrahydrofolate; MTHFR, Methylene Tetrahydrofolate Reductase; CBS, Cystathionine-beta synthase; Cystathionine lyase.

Three enzymes, namely methylenetetrahydrofolate reductase (MTHFR), methionine synthase (MTR) and methionine synthase reductase (MTRR) exert a major role in both Hcy and folate metabolic pathways (Figure 2).

Recently, two frequent sequence variants of the MTHFR gene, the c.677C> T (p.Ala222Val; rs1801133) and the c.1298A > C (p.Glu429Ala; rs1801131), have been associated with a decreased enzymatic activity and increased Hcy concentrations (59, 60). Nelen and coworkers proved that two months of daily supplementation of 0.5 mg folic acid in females with a history of unexplained frequent miscarriages and homozygous for the MTHFR-gene 677C> T mutation reduced their Hcy concentrations (61). Therefore, these studies demonstrate the importance of the nutritional supplementation with folate, especially in infertile patients with MTHFR polymorphisms related to the folate metabolism (61, 62).

### The 1-C Cycle Support (1-CC)

Hcy is metabolized into methionine via the 1-CC. From Hcy, Cysteine can be produced via the cystathionine betasynthase pathway (CBS), a source of the 1-CC, and can be utilized for the synthesis of glutathione and hypotaurine. However, in the oocyte and the early embryo the CBS pathway is not expressed (63). To prevent some birth defects, a supplementation with folic acid is recommended during the preconception period and during the first trimester of pregnancy. Guidelines recommend a daily intake of 400 μg of folic acid during the preconception period and during the first trimester of pregnancy to prevent neural tube defects (NTDs) (64). The MTHFR 677C-T variant, detected with a frequency of up to 15% in female population, encodes for a less efficient protein in Hcy catabolism. Therefore, in patients carrying this variant, folic acid may generate its negative feedback effect, determining a block of the folic acid cycle. Cornet and coworkers reported the beneficial effect of the nutritional support based on supplementation of the 1-CC in subfertile couples having failed previous ARTs (49).

## The Mediterranean Diet (*MedDiet*)

The MedDiet is a healthy eating plan inspired by the nutritional regime of the populations of Greece, Southern Italy, and Spain in the fifties of the twentieth century. The main features of this diet are high amount of legumes, vegetables and fruits, olive oil, unrefined cereals, moderate to high consumption of fish, wine and low intake of meat.

A systematic review and meta-analysis performed in 2011 on 13,800 cancer patients and 23,340 healthy controls reported that adherence to the MedDiet was related to a reduced risk of death from cancer and specifically that the regular consumption of olive oil may reduce the probability of cancer (65).

A 2013 Cochrane review performed on 52,044 randomized participants showed limited positive effects of the MedDiet on cardiovascular risk (66), whereas in the same year a meta-analysis performed on 3,073 randomly assigned individuals with different dietary regimes showed that the MedDiet as well as diets characterized by low-glycemic index, low-carbohydrate and high-protein determined an improvement in cardiovascular risk factors in patients with diabetes (67).

**Figure 3:**
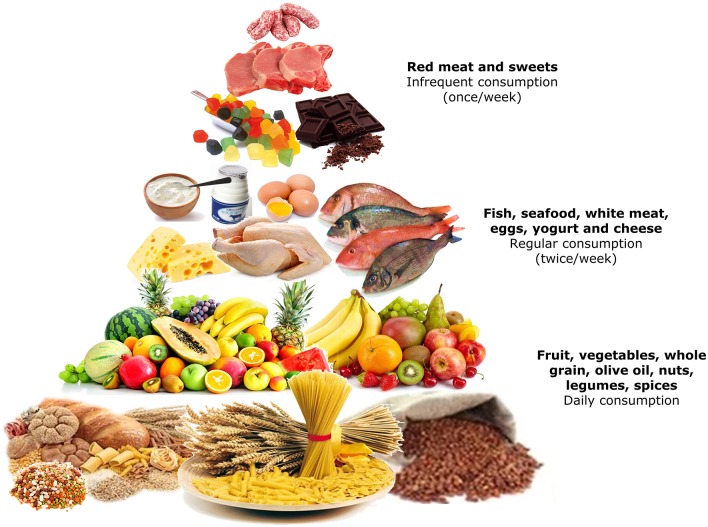
The Mediterranean pyramid of nutrition style and weekly organization of meals.

More recently, a huge umbrella review conducted on more than 12,800,000 subjects from 29 meta-analyses demonstrated a robust statistical association between adherence to the MedDiet and risk reduction of cardiovascular and neurodegenerative diseases, cancer incidence and diabetes (68) (Figure 3).

Recently, several studies showed that different diets in preconception may impact IVF outcomes, and the MedDiet was positively related with folate and vitamin B6 in blood and follicular fluid levels, with a 40% increase in the possibility of pregnancy (36). Karayiannis and colleagues, evaluated the influence of the MedDiet on a better IVF performance in women attempting pregnancy (69) (Table 2) and found that there was no association between MedDiet and the outcomes of IVF or the frequency of implantation. However, MedDiet score was positively related to clinical pregnancy and live birth in particular in women of <35 years old with respect to the older ones (*P* ≤ 0.01). Furthermore, a beneficial 5-point increase in MedDiet score in the same group of women (<35 years), was associated with a 2.7-fold higher probability of achieving clinical pregnancy and live birth, thus proving the efficacy of the MedDiet in preserving the female reproductive health (70). However, the molecular mechanisms underlying both cellular and molecular mechanisms of the relationship between the nutritional regimen and embryo quality, implantation, periconceptional and perinatal development, are presently under intensive investigation.

**Table 2:** Results and recommendations of the Nurses' Health Study II (NHSII) to reduce the risk of infertility due to ovulatory disorders.

	**Risk of ovulatory infertility (*%*)**	**Recommendations NHSII “Mediterranean diet”**
Carbohydrates	1.90	Decrease the glucides at high glycemic
Lipids	1.79	Consume a portion a day of products rich in fat
Protein	1.41 0.78	2% Increase in the intake of polynsatured fatty acids 2%Increase in the intake of monounsatured fatty acids
Folates	0.59	Consume vitamin supplements: B6, B9, and B12
Iron	0.60	Consume nutritional supplements of iron

In a prospective association study on the consumption of polyunsaturated fatty acids, Hammiche F. and collaborators, observed a significant correlation among embryo morphology after IVF and dietary intake of the Omega-3 fatty acids (Fas), Alpha-linolenic acid and Docosahexaenoic acid (DHA) and embryo morphology after IVF (37). Moreover, Yang and coworkers demonstrated that high fat diets resulted in lipid accumulation and endoplasmic reticulum (ER) stress in oocytes in rodents, while the lower folate levels in follicular fluids correlated to poor embryo quality (71). However, to date no large studies have suggested optimized periconceptional diets for fertility outcomes, neither for perinatal or child health outcomes (40).

Other investigators are defining how a modification of the diet for 6 weeks may have favorable effects on developing oocytes and sperms, or for embryo implantation. These authors suggest that blood and follicular biomarkers, useful for evaluating the embryo quality in terms of implantation potential, can be identified and analyzed at the time of oocyte retrieval in IVF (72).

This study also aims to investigate the glucose and the amino acid content of human endometrial fluid, to elucidate whether a diet rich in omega-3 and Vitamin D may significantly improve embryo development. Therefore, this study will be the first prospective randomized controlled trial in humans examining the effects of dietary intervention on the embryo quality (PREPARE Trial) (72). In conclusion, despite the benefits of the MedDiet in both the management and evolution of some clinical conditions and in IVF outcomes, it is still not easy to translate this type of nutrition to daily clinical practice aimed at improving the health conditions (73).

## How to Improve Female Fertility With a Proper Dietary Regimen and Oral Supplementation

Several ovulatory disorders are directly linked to metabolic pathologies such as diabetes and galactosemia, suggesting that dietary factors may play an etiological role in some types of infertility, whereas the role of nutrition in female fertility is still unexplored.

Nevertheless, there are few studies available to date on the role of different nutrients on fertility. Most of the available data are from the Nurses' Health Study cohort and are based on a subsample of 18,555 married women with no history of infertility who desired or achieved pregnancy over an 8-year follow-up period (45). Of these women, 438 reported infertility related to ovulation disorders. In this population, diet was evaluated by a survey on food consumption that provided an estimate of the intake of different nutrients, vitamins, minerals, and fiber. The results showed significative association between female fertility and food behaviors including: consumption of low-glycemic carbohydrates, monounsaturated fatty acids, proteins of plant origin and supplementation with iron, folate and vitamins with antioxidant effect (Table 2). The adherence to this type of MedDiet is associated with a lower risk of infertility related to ovulatory disorders and it has been thus estimated that a healthy diet, combined with a sufficient intake of antioxidants, body weight control and regular physical activity, reduces 69% of the risk of ovulatory infertility. A new topic of increasing interest is the role of the dietary antioxidant consumption, based on the evidence of an experimental correlation between low antioxidant status and known and idiopathic infertility (45). In this contest, Ruder E.H. and coworkers, in their study exploring 437 couples treated for unexplained infertility, showed that a higher ingestion of antioxidants (β-carotene, Vitamins C and E) was related to a shorter time to pregnancy (74).

### The Oral Supplementation Against the Endocrine Disruptors

Animal studies have demonstrated that an appropriate dietary assumption of methyl donor supplements can reduce the effects of environmental endocrine disrupting chemicals (EDCs). EDCs are usually components of cosmetics and “domestic use” products. They induce abnormal effects on methylation profiles and regulatory epigenetic mechanisms in their transgenerational transmission. In animal models, plastic products such as Bisphenol A (BPA), Di(2-ethylhexyl)phthalate (DEHP), and Dibutyl phthalate (DBP) induce transgenerational reproductive and metabolic pathologies. The relation between EDCs, OS, and DNA methylation abnormalities is now well-recognized, namely EDCs generate OS through estrogen receptors and peroxisome proliferator-activated receptors (75).

**Figure 4:**
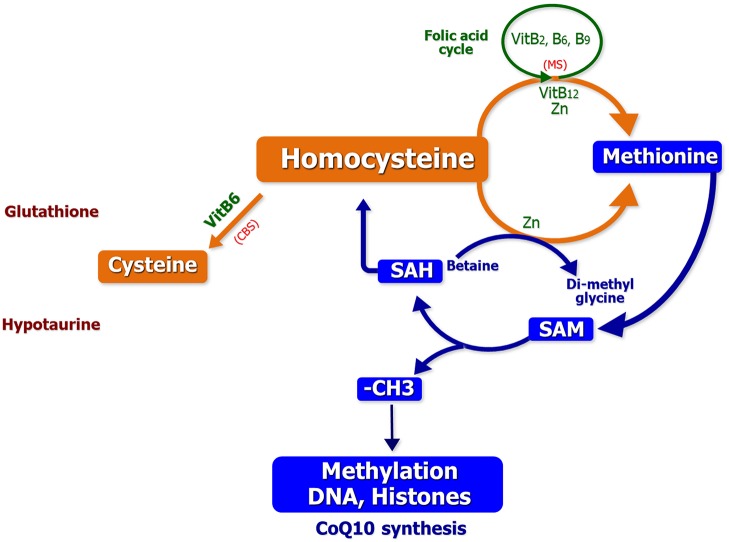
The one carbon cycle: contribution to methylation process and genesis of major antioxidant molecules (76).

BPA has a strong structural similarity with Diethylstilbestrol and E2. Human epidemiological studies have recently highlighted the presence of BPA, parabens, and other organic pollutants in the urine or serum of women who have difficulty in conceiving and/or suffer early menopause. Experiments in the Avy/a (yellow) mouse have shown that methyl donor supplements (support of the 1-CC) definitely affect gene expression and counteract the hypomethylation effects of BPA. As already mentioned above, the 1-CC is involved in the methylation processes, by recycling Hyc, and the generation of endogenous antioxidants such as glutathione and hypotaurine, as well as CoQ10, capable of modulating epigenetic settings in association with Vitamins B2 and B3 (Figure 4) (77). In a recent clinical study, our group proved the efficacy of the 1-CC support in subfertile couples to counteract the negative effects of the environment. In fact, a cohort of 55 women with a history of 3–7 years of sterility, with at least 2 failed ART procedures, and low serum levels of AMH, were treated with 1-CC supplement for 4 months. Surprisingly, we observed 8 spontaneous conceptions within 3 months (76). Therefore, as observed in animal models, an adequate nutritional support of 1-CC can at least in part improve the environmental metabolic derangements causing ovary related infertility (76).

## Conclusions

Female infertility is a global medical and social condition caused by various pathophysiological alterations. In a remarkable number of cases the pathogenesis is not clearly defined, determining indecision concerning the most appropriate treatment choices. While in developing countries this condition is related to preventive, diagnostic and therapeutic inadequacy, multiple ovarian endocrine dysfunctions in industrialized nations are apparently associated with improper life-styles.

In this context, IR appears as a major pathogenic mechanism impairing the physiology of ovulatory functions, while an adequate intake of monounsaturated fatty acids from vegetables may be effective in the prevention of female infertility. Adequate intake of antioxidants also supports female reproductive functions since the dietary supplements containing folic acid, β-carotene, Vitamin C and E, and an adequate nutritional support of the 1-CC are definitely efficient in shortening the time to conception.

In conclusion, a correct balance of proteins, carbohydrates, lipids, antioxidant and folate in the daily diet provides essential benefit for an optimal female reproductive health and reduces the risk of infertility. In this context, the association of MedDiet with antioxidants compounds and 1-CC support appears suitable to improve women's fertility.

## Author Contributions

This review is the result of the contributions of all authors. ES and RP were responsible for literature research, synthesis, and summarization of results. DL analyzed data and drafted the manuscript. ES had primary responsibility for final content. All authors read and approved the final manuscript.

### Conflict of Interest Statement

The authors declare that the research was conducted in the absence of any commercial or financial relationships that could be construed as a potential conflict of interest.
